# Executive control training from middle childhood to adolescence

**DOI:** 10.3389/fpsyg.2014.00390

**Published:** 2014-05-07

**Authors:** Julia Karbach, Kerstin Unger

**Affiliations:** ^1^Department of Educational Science, Saarland UniversitySaarbrücken, Germany; ^2^Department of Cognitive, Linguistic, and Psychological Sciences, Brown UniversityProvidence, RI, USA

**Keywords:** executive control, cognitive training, childhood, adolescence, cognitive plasticity

## Abstract

Executive functions (EFs) include a number of higher-level cognitive control abilities, such as cognitive flexibility, inhibition, and working memory, which are instrumental in supporting action control and the flexible adaptation changing environments. These control functions are supported by the prefrontal cortex and therefore develop rapidly across childhood and mature well into late adolescence. Given that executive control is a strong predictor for various life outcomes, such as academic achievement, socioeconomic status, and physical health, numerous training interventions have been designed to improve executive functioning across the lifespan, many of them targeting children and adolescents. Despite the increasing popularity of these trainings, their results are neither robust nor consistent, and the transferability of training-induced performance improvements to untrained tasks seems to be limited. In this review, we provide a selective overview of the developmental literature on process-based cognitive interventions by discussing (1) the concept and the development of EFs and their neural underpinnings, (2) the effects of different types of executive control training in normally developing children and adolescents, (3) individual differences in training-related performance gains as well as (4) the potential of cognitive training interventions for the application in clinical and educational contexts. Based on recent findings, we consider how transfer of process-based executive control trainings may be supported and how interventions may be tailored to the needs of specific age groups or populations.

## INTRODUCTION

Over the last decade, the scientific interest in cognitive interventions designed to improve cognitive functions in childhood and adolescence has been rapidly increasing. The many studies investigating the benefits of cognitive training interventions showed that cognitive plasticity is considerable not only in children and adolescents, but also up to old age (for recent reviews, see Buitenweg et al., 2012; Diamond, 2012; Karbach and Schubert, 2013; Kray and Ferdinand, 2013; Strobach et al., 2014; Titz and Karbach, 2014; Verhaeghen, 2014). These studies usually showed significant performance improvements on the trained tasks. Moreover, they oftentimes also revealed near transfer to tasks that were not explicitly trained but measured the same construct as the training task, and sometimes even far transfer to tasks measuring a different construct.

Despite these encouraging findings, the literature clearly shows that these transfer effects were not consistent across studies, a fact that has inspired intense recent debates regarding the transferability of training-induced performance gains (e.g., Shipstead et al., 2012; Melby-Lervåg and Hulme, 2013; Redick et al., 2013). The inconsistent pattern of results may be explained by the large differences in terms of the type, intensity, and duration of the training regimes and the fact that different methodologies haven been adopted across studies. Thus, the comparability of previous results is often very limited.

In addition, it makes sense to differentiate different types of cognitive training interventions: strategy-based training refers to interventions involving the training of task-specific approaches designed to support the execution of certain tasks. It has often been applied in memory training studies and typical examples include mnemonic techniques, such as the method of loci. This type of memory strategy training often resulted in large and often long-lasting improvements on the training task, but induced only limited transfer (for meta-analyses, see Verhaeghen et al., 1992; Rebok et al., 2007). Multi-domain training interventions are usually more complex and engage multiple cognitive processes (e.g., game-based training), yielding broad but often small transfer effects (e.g., Basak et al., 2008). The main disadvantage of multi-domain trainings is that their complex nature makes it hard to determine which specific features of the training regime induced transfer.

In contrast, process-based training protocols are not task-specific because they target more general processing capacities supporting a range of cognitive operations, such as speed of processing or executive functions (EFs). Some process-based interventions, mainly from the domain of EF, have resulted in very promising widespread transfer across the lifespan (Hertzog et al., 2008; Karbach and Schubert, 2013; Kray and Ferdinand, 2013; Titz and Karbach, 2014), suggesting that process-based training might be more efficient than strategy-based interventions. The fact that EF may be improved by means of cognitive training is of particular importance in childhood and adolescence, because EF is a strong predictor for various life outcomes, such as academic attainment, socioeconomic status, and physical health (e.g., Eigsti et al., 2006; Blair and Razza, 2007; Moffitt et al., 2011). Moreover, behavioral and neural plasticity is particularly high in childhood and the brain areas serving EF (i.e., the prefrontal lobes) are especially sensitive to environmental influences in children (cf. Bull et al., 2011). It is therefore not surprising that numerous training interventions have been designed to improve executive functioning across the lifespan, many of them targeting children and adolescents. These studies have included normally developing children as well as individuals suffering from neurodevelopmental or psychiatric disorders, some of which are characterized by significant cognitive deficits [e.g., attention-deficit hyperactivity disorder (ADHD) or autism].

A number of recent systematic reviews and meta-analyses have focused on training interventions targeting EF in children. Some of them have analyzed findings from samples with cognitive impairments (e.g., Rapport et al., 2013; Chacko et al., 2014), others have selectively focused on specific types of training (e.g., Kray and Ferdinand, 2013) or on specific methodological approaches, such as neuroscientific techniques (e.g., Buschkuehl et al., 2012; Jolles and Crone, 2012), or on specific outcome measures, such as academic achievement (e.g., Titz and Karbach, 2014). Comprehensive reviews including training on different components of EF in samples of normally developing children have been focused on preschoolers (e.g., Diamond, 2012; Zelazo and Lyons, 2012) and so far a systematic review of recent findings on EF training in middle childhood and adolescence is still missing. Such a review may contribute to the understanding of the cognitive mechanisms underlying plasticity of cognitive functions across their development in middle childhood and adolescence. Considering the importance of EF for numerous life outcomes, the identification of successful cognitive training interventions may not only be beneficial for the compensation of cognitive deficits in clinical samples, but also to promote cognitive performance and development in healthy children and adolescents.

Therefore, the aim of this review is to (1) illustrate the concept of EF, its neural correlates and age-related changes in middle childhood and adolescence as an introduction to (2) the presentation of selected recent findings of process-based EF training in this age group, followed by (3) the description of individual differences in training-related improvements. We close by (4) outlining potential applications of EF training in clinical and educational settings.

### DEFINITIONS OF EF

The term executive control refers to a broad collection of higher-order cognitive functions that allow individuals to flexibly regulate their thoughts and actions in the service of adaptive, goal-directed behavior. EFs are typically thought to encompass a wide range of mental processes that vary in complexity and abstractness, such as working memory, cognitive flexibility, attentional control, planning, concept formation, or feedback processing (e.g., Jurado and Rosselli, 2007). Working memory serves to update and monitor information and to code task-relevant information. This relevant information is held in working memory until it is no longer needed and subsequently replaced with newer, more relevant information. Working memory is required to mentally relate, integrate, and recombine information across different time scales and hence plays a pivotal role for more complex EFs such as planning or concept formation. Following a conversation in a foreign language puts high demands on working memory resources, as does difficult mental arithmetic or planning the optimal route from city center to airport during rush hour traffic. Also referred to as *shifting*, *attention switching*, or *task switching*, cognitive flexibility refers to the ability to flexibly shift between tasks, goals, or mental sets. It involves disengaging from currently irrelevant information (i.e., the previous task set) and focusing on currently relevant information (i.e., the upcoming task; e.g., Meiran, 1996; Monsell, 2003). Cognitive flexibility allows us to think divergently and creatively and to respond quickly to unpredicted changes in the environment. It helps us to change the perspective and develop new solution ideas when we are stuck with a problem (e.g., trying to handle a new electronic device or software tool) or to use unexpected opportunities such as backing up in a parking spot that suddenly opens up behind us while we are waiting for another car to leave a parking space in front of us. Attentional control is required when we need to focus on a specific stimulus while minimizing interference from irrelevant stimuli. In everyday life, we use this ability when we are talking on the phone and have to tune out conversations of other people around us. Another form of control involves the inhibition of automatic or impulsive response tendencies and unwanted emotions. For example, if a deer suddenly jumps out in front of our car, we have to suppress the tendency to swerve. Similarly, if we want to lose weight, we have to resist sweets and fatty foods and social norms dictate us not to yell at another person even if we are angry.

The heterogeneity of the processes described above highlights the need to determine the structure and organization of executive control more precisely. A key question in this context was whether EFs are best characterized as unitary or multi-dimensional in nature. Early theoretical frameworks mostly adopted the perspective that a common cognitive mechanism or ability underlies executive functioning. Prominent examples are Norman and Shallice’s (1986) “Supervisory Attentional System” or the closely related “Central Executive” in Baddeley’s (1986) working memory model. A more recent proposal by Duncan et al. (1996) established a theoretical link between the concept of a prefrontally based unitary control system and Spearman’s general intelligence factor *g* (see also Denckla and Reiss, 1997; Kimberg et al., 1997; de Frias et al., 2006). In a similar vein, Salthouse (2005) noted that interindividual differences in executive functioning may tap basic reasoning skills and perceptual speed (but see Ardila et al., 2000; Friedman et al., 2006). Empirical support for the unitary nature of executive control comes from psychometric studies showing that different components are substantially correlated at the latent variable level (e.g., Miyake et al., 2000; Friedman et al., 2008, 2011). The intercorrelations among these latent factors, however, are usually moderately high, indicating that EFs comprise clearly separable subcomponents even though they may share some commonalities.

In an influential study, Miyake et al. (2000) provided first conclusive evidence for the “unity/diversity” framework by applying a latent variable approach to a task battery designed to capture three putative core components of executive control (see above for a more detailed description of the involved processes): (1) flexibly switching between different task sets or mental representations (*shifting*), (2) updating, removing, and monitoring working memory contents (*updating*), and (3) overriding prepotent response tendencies and suppressing attention to irrelevant stimuli as well as unwanted thoughts and emotions (*inhibition*; Miyake et al., 2000). The authors demonstrated that a full three-factor model that allowed correlations between the three latent variables yielded a better fit than either a three-factor model that did not allow for such correlations or any other single- or two-factor model. Interestingly, subsequent work showed that when variance common to all executive tasks was accounted for by a unity factor (referred to as common EF), only the shifting and updating factors captured unique variance (Friedman et al., 2011). Thus, there was no evidence for an inhibition-specific ability that is separable from the common factor. As Miyake and Friedman (2012) pointed out, a strong candidate mechanism for this common basic ability is the stable maintenance of task goals and goal-relevant representations in working memory, whereas the updating- and shifting-specific component might reflect effective gating and clearance of those representations (Herd et al., submitted). Specifically, it has been hypothesized that updating might be associated with efficient gating of information into working memory and/or controlled long-term memory retrieval (Miyake and Friedman, 2012). The shifting-specific component, in contrast, has been suggested to reflect mental “stickiness” (Altamirano et al., 2010; Herd et al., submitted), denoting the uncontrolled, automatic persistence of goal representations that are no longer relevant and hence should be removed from working memory.

### NEURAL UNDERPINNINGS OF EF

Historically, the study of the neural substrates underpinning EFs originated from the observation of common deficits in patients with frontal lobe lesions (Stuss and Benson, 1986), including impairments in working memory, planning, and inhibition (Shallice and Burgess, 1991). Although the prefrontal cortex (PFC) is thought to play a key role in mediating executive control, neuroimaging and lesion studies demonstrated that the performance of executive tasks is associated with activation in a large set of brain regions, involving prefrontal and parietal areas, motor regions, as well as subcortical structures, such as basal ganglia and thalamus (Duncan and Owen, 2000; Dosenbach et al., 2008; Niendam et al., 2012).

In line with the unity/diversity framework, a number of reviews and meta-analyses demonstrated that performance on different EF tasks reflects the joint contribution of a common frontoparietal network and unique, component-specific brain regions (Wager and Smith, 2003; Wager et al., 2004; Collette et al., 2006; Niendam et al., 2012). Specifically, it has been shown that shifting, updating, and inhibition tasks elicit overlapping activation in frontal [e.g., dorsolateral PFC (DLPFC), the anterior cingulate cortex (ACC)] and parietal regions (e.g., superior and inferior parietal lobe, precuneus) associated with the common executive control network. Component-specific (i.e., non-overlapping) activations were observed in distinct prefrontal, occipital and temporal areas (including BAs 6, 10, 11, 19, 13, and 37). Furthermore, analyses showed unique activation patterns in subcortical regions, including caudate, thalamus, putamen, and cerebellum, for inhibition and updating tasks.

Similar conclusions have been drawn in a positron emission tomography (PET) study by Collette et al. (2005) that used conjunction analyses to identify common neural substrates of the executive tasks administered by Miyake et al. (2000). Findings revealed that left superior parietal gyrus, right intraparietal gyrus, right intraparietal sulcus and, albeit less robustly, left middle and inferior frontal gyri were commonly engaged by all three executive processes. Although pairwise comparisons of the specific component processes showed dissociations in frontoparietal activation patterns, the observed differences do not easily map onto the latent factor structure suggested by Miyake et al. (2000) and Miyake and Friedman (2012).

Consistent with Miyake et al.’s (2000) proposal, the common frontoparietal network, especially the prefrontal part, is thought to play a major role in actively representing and maintaining task-goals, task context or task sets (rules) in order to bias down-stream information processing (Miller and Cohen, 2001; Rossi et al., 2009). Munakata et al. (2011) recently proposed a framework that describes how inhibitory control emerges from this key function of the PFC. Specifically, the authors argue against the widely held view that certain prefrontal regions, such as the right inferior frontal gyrus, are functionally specialized to subserve inhibition. Instead, the framework posits that specific contributions of different prefrontal regions to inhibitory processes depend on the kind of information they represent and their interconnections with other brain areas. Thus, for instance, the ACC and related medial frontal areas are thought to use signals of conflict, errors, or uncertainty to inhibit inappropriate motor responses via projections to the subthalamic nucleus.

A prevalent view is that parietal regions such as intraparietal sulcus or inferior parietal lobule are involved in the top-down control of attention (Corbetta and Shulman, 2002) and may support executive control by subserving functions such as cue decoding or signaling of stimulus conflict (Dosenbach et al., 2008). In addition, parietal activation has been linked to maintenance of stimulus–response (S–R) mappings (Bunge, 2004) well as manipulation of working memory contents (Wendelken et al., 2008).

Furthermore, accumulating evidence indicates that EFs critically rely on complex interactions between PFC and subcortical structures via frontal corticobasal ganglia and frontal corticocerebellar circuits (e.g., Heyder et al., 2004; Gruber et al., 2006; O’Reilly and Frank, 2006). For instance, Miyake and Friedman (2012) suggested that updating might be associated with selective and efficient gating of information into working memory via corticostriatal loops. In addition, Herd et al. (submitted) used a computational modeling approach to explore two potential neural mechanisms underlying individual differences in the shifting-specific component of executive control: (1) recurrent connection strength in PFC and (2) efficient clearing of old representations from working memory upon gating decisions from the basal ganglia. Both are thought to influence the tendency to maintain information in working memory that is no longer task-relevant.

### AGE-RELATED CHANGES IN EF

Infant research has shown that elementary forms of executive control emerge within the first year of life (Carpenter et al., 1998; Diamond, 2006). Although core components of executive control, including working memory, inhibition and attentional flexibility, can be observed in preschoolers as young as 3 years of age (Hughes, 1998), EFs continue to improve throughout childhood into late adolescence or even adulthood (Davidson et al., 2006; Huizinga et al., 2006; Diamond, 2013). In recent years, a number of studies have addressed the key question of whether the unity/diversity framework appropriately describes the structure of EFs in children and adolescents (e.g., Lehto et al., 2003; Gathercole et al., 2004; Huizinga et al., 2006; Wiebe et al., 2008, 2011; Rose et al., 2011). Most findings support the notion that the latent factor structure of executive control changes qualitatively across development, from a unitary structure (i.e., a single-factor structure) in preschoolers to multiple subcomponents in school-age children and adolescents.

Developmental trajectories of EFs are thought to be inextricably linked to maturational changes of prefrontal regions and associated cortical and subcortical structures, including parietal regions and basal ganglia (e.g., Casey et al., 2005; Bunge and Wright, 2007; Luna et al., 2010). Behavioral improvements in cognitive control coincide with synaptic pruning and increased myelination as well as experience-dependent synaptic strengthening (Sowell et al., 2001; Bjorklund, 2005; Dawson and Guare, 2010). Some regions within PFC, such as orbitofrontal cortex, reach structural maturity at an earlier age, whereas others, such as DLPFC, show more protracted maturational time course (Gogtay et al., 2004). There is evidence to suggest that those differences in structural maturation are paralleled by changes in functional maturation and hence may account for distinct developmental trajectories among EFs (Bunge and Zelazo, 2006). Moreover, it has been demonstrated that there are substantial developmental changes in the structure of neural network(s) underlying executive control (Fair et al., 2008), with the number of short-range connections decreasing (segregation) and the number long-range connections increasing (integration) from childhood to adulthood. Using neural network modeling, Edin et al. (2007) showed that age-related differences in activation in the superior frontal sulcus and intraparietal sulcus during working tasks can be accounted for by stronger fronto-parietal (i.e., interregional). In contrast, stronger intraregional connectivity as well as faster conduction or increased coding specificity could not explain developmental changes in patterns of brain activity. In the following, we outline major developmental changes of the three core EFs cognitive flexibility/shifting, working memory/updating, and inhibition.

The ability to flexibly shift between task sets shows the most protracted development and continues to improve into adolescence (Chevalier and Blaye, 2009; Best and Miller, 2010; Diamond, 2013). Although 3- and 4-year-old children are able to successfully shift between two simple rules (e.g., Zelazo, 2004; Moriguchi and Hiraki, 2009, 2011), performance continues to improve at later ages for more complex task sets and higher numbers of rules. Several studies have consistently shown that two components of task shifting – the ability to switch from one rule to another rule (i.e., switching *per se*) and the ability to maintain and select two (or more) rules – follow different developmental time courses (e.g., Crone et al., 2004, 2006; Kray et al., 2004, 2008, 2012; Huizinga and van der Molen, 2007; Karbach and Kray, 2007). For instance, Huizinga and van der Molen (2007) reported that children’s set switching abilities reached adult levels by the age of 11 years, whereas set maintenance continued to improve by the age of 15 years. Moreover, findings by Crone et al. (2006) indicated that the different developmental trajectories of rule representation/retrieval and rule switching/suppression are associated with differences in the recruitment of ventrolateral PFC (VLPFC) and pre-supplementary motor area (pre-SMA/SMA), respectively. Another study on task switching (Rubia et al., 2006) found age-related increases in the recruitment of several brain regions that have been implicated in shifting, including right inferior PFC, left parietal cortex, ACC, and striatum. In contrast, Wendelken et al. (2012) observed similar activation of frontoparietal control regions across children and adults during task switching.

While basic updating processes can be observed in 9- to 12-month-old infants, the ability to manipulate items in working memory develops later and over a longer time range (Diamond, 2013). Working memory performance at more complex tasks has been shown to improve linearly from pre-school age to adolescence (Gathercole et al., 2004), with age differences varying as a function of complexity (Luciana et al., 2005). Developmental neuroimaging studies typically focused on simple maintenance demands (Bunge and Wright, 2007). These studies revealed a complex pattern of age-related changes in brain activation. Some of the regions that have been associated with working memory processes in adults, such as the superior frontal sulcus and the intraparietal sulcus, show activation increases across childhood and adolescence, whereas others, such as the DLPFC and parietal cortices, are recruited to a lesser degree. There is also evidence for qualitative changes in neural activation. Scherf et al. (2006) observed that children engaged a compensatory network, including caudate nucleus, anterior insula, and lateral cerebellum, whereas adolescents recruited an adult-like working-memory circuitry comprising core structures like DLPFC and ACC, albeit to a lesser degree. Findings from other studies indicated that children are less able to suppress interference (Kray et al., 2012). Furthermore, Crone et al. (2006) provided evidence that children’s performance deficits at tasks that require manipulation of information in working might be related to their failure to recruit frontoparietal regions.

Inhibitory control develops rapidly during the preschool years and typically continues to improve into middle childhood (Kray et al., 2009, 2012; Best and Miller, 2010). However, some studies using computerized tasks reported continued improvement until adolescence or even young adulthood (e.g., Huizinga et al., 2006). Developmental trajectories have been found to depend strongly of the nature of the inhibition task, suggesting that different tasks tap into distinct control processes (Nigg, 2000). Similar to the developmental course of shifting and working memory, age differences in inhibition vary as a function of task (rule) complexity (Zelazo, 2006). Depending on the specific inhibitory requirements of the task, the ability to override a prepotent response has been found to improve most rapidly between ages 3 and 4 years (Hughes, 1998) or – particularly when the task involves concurrent working memory demands, the response bias is stronger, or responses have to be inhibited at a late stage (of execution) – between ages 5 and 8 years (Romine and Reynolds, 2005). Best and Miller (2010) suggested that early improvements in inhibitory control mainly reflect qualitative changes in information processing such as children’s conceptual understanding of the hierarchical rule system underlying tasks like the dimensional change card sort (DCCS; Zelazo, 2006), while later improvements indicate quantitative changes, such as increasing efficiency of the underlying cognitive mechanism. Neuroimaging studies established a functional link between recruitment of right VLPFC as well as functionally connected subcortical areas such as thalamus, nucleus caudate, cerebellum, and the development of mature response control (Rubia et al., 2001; Bunge et al., 2002). Moreover, evidence from EEG studies indicated that refinements in stimulus processing (e.g., better stimulus discrimination) contribute to age-related performance increments on inhibitory control tasks (e.g., Johnstone et al., 2007).

## TRAINING EF IN HEALTHY INDIVIDUALS

Given that EFs are subject to significant developmental progress across childhood and up to late adolescence and because they are significant predictors for many life outcomes (see Introduction), numerous studies aimed at improving these control functions by means of cognitive training interventions. Even though most of these studies have been restricted to the assessment of cognitive abilities via experimental training and transfer tasks at the lab, their ultimate goal is to improve children’s EF in order to facilitate typical activities in their daily lives, such as learning and academic development (for a review, see Titz and Karbach, 2014). Even though evidence for the transfer of EF training to activities of daily living is still limited (see Potential for the Application in Clinical and Educational Settings), the existing studies have provided important insights into the mechanisms underlying behavioral plasticity (see Training EF in Healthy Individuals) and their neural underpinnings. In keeping with Miyake et al.’s (2000) model of EF, we selectively review studies that have trained cognitive flexibility/shifting, working memory/updating, and inhibition in school-aged children and adolescents.

### COGNITIVE FLEXIBILITY/SHIFTING

While studies investigating cognitive flexibility in preschoolers have often applied card sorting tasks such as the DCCS (Zelazo, 2006), training studies including children over the age of 6 years have mostly relied on task-switching training in order to improve cognitive flexibility. In task-switching studies, participants are instructed to perform two or more simple decision tasks and to switch between them upon a specific cue or in a specific order. For instance, they may be required to decide whether a picture presented on the computer screen shows a fruit or a vegetable (task A) on some trials and to decide whether the picture is small or large (task B) on other trials (cf. Karbach and Kray, 2009). Comparing performances in task-homogeneous blocks (only task A or task B has to be performed) to performances in task-heterogeneous blocks (participants have to switch between task A and B) allows assessing the ability to maintain and select two task sets (general switch costs). Comparing the performances on switch trials (AB, BA) to performances on stay trials (AA, BB) provides a measure for cognitive flexibility (specific switch costs; for a review, see Monsell, 2003).

While previous studies have shown that task maintenance and selection as well as cognitive flexibility improved after task-switching training in children and adolescents between the ages of 7 and 20 years (e.g., Cepeda et al., 2001; Kray et al., 2008, 2013; Karbach and Kray, 2009; Zinke et al., 2012), there is also evidence for transfer of task-switching training to new untrained tasks. When it comes to childhood, a study including a sample of children between the ages of 7 and 9 years as well as younger adults and older adults (*N* = 168), investigated the effects of four sessions of intensive internally cued task-switching training. Compared to a control condition including the same tasks without the switching component (single-task training), the task-switching training resulted in performance improvements on a structurally similar untrained switching task (near transfer), as well as measures of inhibition, verbal and visuo-spatial working memory and reasoning (far transfer) (Karbach and Kray, 2009; see also Kray et al., 2012). The findings of this study showed that the transfer of task-switching training was not merely mediated by an automatization of the two component tasks A and B (which were also trained in the control condition). Moreover, a comparison of different training conditions showed that children’s transfer was reduced when the training tasks were different switching tasks in each one of the four training sessions (as compared to the same task in each session), while the opposite pattern was found in adults. Thus, the increased cognitive load associated with the need to adapt to new training tasks in each session may not have left enough processing capacity to implement the trained abilities and to develop cognitive representations of the task structure (cf. van Merriënboer et al., 2006).). One interesting finding was the broad scope of transfer, especially considering that transfer in many other training studies was more limited (see Shipstead et al., 2012; Melby-Lervåg and Hulme, 2013; Redick et al., 2013). The authors attributed the transfer to the nature of the training task, which required a number of EF abilities: demands on goal maintenance and task set-selection were high during training, because participants had to maintain the task sequence, as they did not receive external task cues. Moreover, inhibitory control was required at all times because the stimuli were ambiguous (i.e., they always represented features relevant for both component tasks). Therefore, the training of multiple EF abilities may have supported transfer to other executive and cognitive tasks.

A similar training regime was investigated in a sample of adolescents (10–14 years of age, *N* = 80). In this study, the task-switching training was compared to a passive control group, a physical exercise group and a group performing task-switching training and physical training (Zinke et al., 2012). Analyses showed a reduction of specific switch costs over the course of the training. These improvements were driven by larger training-related benefits on switch trials as compared to stay trials, suggesting that the training specifically benefited the ability to switch between tasks and not merely increased the general speed of response execution. Interestingly, three sessions of task-switching training yielded performance benefits on an untrained switching task (near transfer) and in terms of choice reaction time and updating, but not inhibition (far transfer), without additional benefits of the acute boosts of exercise before training. Thus, these results based on adolescent participants were generally consistent with those from children and adults, indicating that task-switching training improved cognitive flexibility and transferred to tasks assessing other dimensions of EF, particularly working memory. The fact that transfer in adolescents was less pronounced than in other age groups may be due to small changes in the training regimen (e.g., the reduction of training sessions), but it may also reflect different developmental trajectories in adolescence (cf. Huizinga et al., 2006) rendering individuals more or less amenable to the effects of cognitive training than other age groups (Zinke et al., 2012).

### WORKING MEMORY/UPDATING

For the assessment of children, one prototypical working memory task is the single *n*-back task, including the presentation of sequences of stimuli, such as digits or pictures. Participants are instructed to respond if the current stimulus matches the one presented *n* trials earlier in the sequence (e.g., Jonides and Smith, 1997). Another frequently applied type of task to assess working memory is the working memory span task. The simple version assesses the maximum capacity of items that can be held in working memory, for instance by instructing participants to remember sequences of digits or spatial positions. In complex working memory span tasks, this memory task has to be performed against a background processing task, such as counting or reading (e.g., Oberauer et al., 2003; Kane et al., 2004).

Given that working memory deficits are associated with a number of developmental disorders and learning difficulties, such as ADHD, dyslexia, and dyscalculia (e.g., Barkley, 1997; Schuchardt et al., 2008) many training studies have focused on clinical samples (such as, for instance, CogMed training; see Potential for the Application in Clinical and Educational Settings) and studies assessing the effects of process-based working memory training on healthy children and adolescents are surprisingly scarce. Jaeggi et al. (2011) assessed the effects of 4 weeks (≈19 sessions) of visuo-spatial *n*-back training in a sample of elementary and middle school students (mean age = 9 years, *N* = 62). Compared to a control group that performed knowledge and vocabulary-based tasks, the working-memory training group showed significant improvement on the training tasks, but no transfer to measures of fluid intelligence. Interestingly, a comparison between the participants that showed large improvements on the training task and those that only displayed small benefits revealed a differential pattern of results regarding the transfer gains: improvements on the training tasks were positively correlated with transfer gains in terms of fluid intelligence, and only the group with large improvements on the training tasks showed significant transfer of the working-memory training to measures of fluid intelligence (matrix reasoning). These differences were found immediately after training as well as three months later. The authors concluded that transfer of working memory training to fluid intelligence depends on participant’s individual improvement on the training task. Moreover, they found that the perceived difficulty of the training task negatively affected training-related gains, pointing to the importance of adaptive task difficulties that are optimally challenging at all times (Jaeggi et al., 2011).

Complex working memory span tasks have been applied in two recent training studies (Loosli et al., 2012; Karbach et al., 2014). Both studies included adaptive training on child friendly tasks from the Braintwister working-memory training battery (Buschkuehl et al., 2008). In these tasks, participants were to remember the sequence of animal pictures (memory task) while analyzing the orientation of each presented picture (processing task), so that successful task execution mainly relied on verbal working memory processes. One of the studies compared the training to a passive control condition (Loosli et al., 2012; *N* = 40, 9–11 years of age; 10 sessions of training) and the other one to an active control group performing a non-adaptive low-level version of the training tasks (Karbach et al., 2014; *N* = 28, 7–9 years of age; 14 sessions of training). The results of both studies were very consistent with respect to reliable performance improvements on the training tasks in the training group and in terms of far transfer to reading abilities (for details, see Potential for the Application in Clinical and Educational Settings). Despite near transfer to a new, untrained working memory task (Karbach et al., 2014), no further transfer to any other experimental tasks occurred across studies, including measures of cognitive flexibility, inhibition, and fluid intelligence. Compared with Jaeggi et al.’s (2011) findings, this data suggests that visuo-spatial working-memory training might be more effective in order to induce transfer to other domains of EF and other cognitive abilities than verbal working-memory training. One line of evidence supporting this idea is the literature on the broad transfer of CogMed working-memory training, which includes both verbal and visuo-spatial training tasks (Klingberg, 2010; for details, see Potential for the Application in Clinical and Educational Settings). However, a systematic comparison of verbal and visuo-spatial working-memory training and the subsequent transfer effects is needed to test this hypothesis.

Moreover, transfer of working-memory training fits well with recent findings from neuroimaging studies on adults. Training on updating and switching tasks (Dahlin et al., 2008; Karbach and Brieber, 2010) has been shown to reduce activity in fronto-parietal networks and increase activity in the striatum (see also Olesen et al., 2004), a structure that is of particular importance for learning processes. It serves as a gating mechanism that decides which processes need to be worked on by the frontal and parietal areas of the brain. Thus, this increased activity in the striatum and, at the same time, the decreased fronto-parietal activation may be indicative of more automated task processing after the training and may suggest a shifted from a broad, dispersed network to a specific and more optimal one mediating efficient executive control processes.

In sum, recent findings from childhood and early adolescence showed that working-memory training has the potential to improve both verbal and visuo-spatial working memory ability (i.e., performance gains on the trained tasks). Evidence for near and especially far transfer of working-memory training to untrained tasks and abilities has been reported less consistently and has been discussed very controversially (e.g., Shipstead et al., 2012; Redick et al., 2013). It seems to depend on the nature of the training, the transfer tasks, and the control condition as well as the baseline performance and the motivation of the participants (e.g., Jaeggi et al., 2011, 2014; Shah et al., 2012; Green et al., 2013; Titz and Karbach, 2014). Results from studies on adults suggest, for instance, that the updating component of working memory (e.g., storage and processing) has to be engaged during training in order to induce transfer to untrained working memory tasks and reasoning (von Bastian and Oberauer, 2013; see also Zinke et al., 2014).

### INHIBITION

A widely used inhibition task is the Stroop task (Stroop, 1935), that requires participants to respond to the font color of words. The stimuli are either congruent (e.g., GREEN in green font color) or incongruent (e.g., GREEN in blue font color). Responses to incongruent stimuli are usually slower and more erroneous than to congruent stimuli (Stroop effect) reflecting the cognitive effort associated with the need to overcome the tendency to produce the more automated action of reading the word instead of naming the font color. Interference control is often assessed by means of the Flanker task (Eriksen and Eriksen, 1974) that requires participants to respond to a stimulus that is flanked by two other stimuli on each side. These stimuli can be congruent (e.g., HHHHH) or incongruent (e.g., SSHSS). Again, responses to incongruent stimuli are typically slower and more erroneous than to congruent stimuli (Flanker effect) reflecting the difficulty of focusing on the stimulus in the middle while suppressing interference from the surrounding letters.

However, even though it has been reported that inhibitory skills could be trained in preschoolers (e.g., Thorell et al., 2009), studies incorporating typical inhibition tasks into their training regimes for older children are rare. To our knowledge, there is no report of a training exclusively relying on inhibition tasks. Still, the training battery applied by Rueda et al. (2005) included a number of tasks tapping stimulus discrimination, conflict resolution, and inhibition, but also visual attention and anticipation exercises. The authors investigated the effects of five sessions of this “executive attention” training in 4- and 6-year-olds. After training, there was no significant near transfer to interference control (measured by the Attention Network Test, a child-friendly version of the Flanker paradigm) but far transfer to intelligence test scores, especially on the matrices scale. Thus, the training battery did not benefit EF, but reasoning abilities in preschoolers and first graders.

Even though explicit inhibition trainings are so scarce, it should be noted that trainings from the domain of working memory and cognitive flexibility often implicitly trained a fair amount of interference control. The task switching studies described above (Karbach and Kray, 2009; Zinke et al., 2012), for instance, included ambiguous stimuli (i.e., stimuli representing features that were relevant for both tasks, such as a large fruit or a small vegetable, for instance) and therefore the need to suppress interference from the currently irrelevant dimension (e.g., “large” when the currently relevant task was to decide between fruit or vegetable) and to focus on the relevant dimension. Moreover, the complex working memory tasks applied in other studies (e.g., Loosli et al., 2012; Karbach et al., 2014) included high demands on inhibitory control because participants had to inhibit the responses from the concurrent processing task in order to properly focus on the memory task: for instance, in the Braintwister trainings tasks, the children were to ignore their responses regarding the orientation of the animals (processing task) and to focus or their sequence (memory task).

Whether and to what extent inhibitory abilities may be improved in older children and adolescents remains to be examined. Future studies may rely on inhibition tasks, such as the Stroop-task, the stop-signal task, or antisaccade tasks, or on interference control tasks, such as the Flanker task. Given that recent studies showed that inhibitory control in adolescents may be improved considerably by motivational factors, such as performance-related rewards (Kohls et al., 2009; Geier et al., 2010), the room for improvement may be substantial.

Thus, recent findings from process-based EF training indicate that each one of the key domains of EF can be improved by cognitive training in childhood and adolescence. There is also evidence for transfer of EF training to other dimensions of EF (e.g., from task-switching training to working memory abilities), supporting the view that executive control is a multifaceted construct including a number of correlated but separable control dimensions (e.g., Miyake et al., 2000; Miyake and Friedman, 2012). The fact that EF training also benefited performance on fluid intelligence tasks (especially matrix reasoning) is consistent with recent latent variable approaches that confirmed a strong relationship between both domains in childhood and adulthood (e.g., Friedman et al., 2006; Engel de Abreu et al., 2010). Future studies need to shed light on specific features of the training regimes and characteristics of the participants that have the potential to support positive effects of cognitive training interventions. This will probably include a shift from the general question of whether a given training in effective or not (i.e., the comparison of mean group differences) to more fine grained analyses testing individual differences in order to determine for whom the training actually works.

## INDIVIDUAL DIFFERENCES IN TRAINING-INDUCED GAINS

The selected findings reviewed above, along with numerous other studies from the field of cognitive training research (for reviews, see Hertzog et al., 2008; Lustig et al., 2009; Noack et al., 2009; Karbach and Schubert, 2013; Kray and Ferdinand, 2013; Titz and Karbach, 2014), showed that cognitive training interventions have the potential to yield significant EF benefits and even transfer of EF training at the group level, but evidence on individual differences is still limited, especially in childhood and adolescence. This is particularly critical in populations displaying rapid cognitive developmental progress, because children and adolescents are likely to differ more from each other than young adults and between-group comparisons do little justice to individuals’ strengths and weaknesses. Therefore, the question who benefits most from cognitive interventions has been more and more acknowledged in the field of cognitive training research lately and an increasing number of studies have analyzed why some individuals benefited more than others. The importance of this question is obvious from an applied point of view, especially when it comes to the adaptation of training interventions to populations with specific needs, such as students with cognitive or academic deficits. Moreover, it also is of interest on the theoretical level, because individual differences in training-related benefits may help us understand the underpinnings of cognitive and neural plasticity.

Two prominent accounts haven been put forward to describe and explain individual differences in training-related performance gains: first, the magnification account (also known as Matthew effect or scissor effect) assumes that individuals that are already performing very well will also benefit most from cognitive interventions. It is assumed that high-performing and well-educated participants have more efficient cognitive resources to acquire and implement new strategies and abilities. Thus, baseline cognitive performance at pretest should be positively correlated with the training-related gains. And with respect to EF, which gradually develop across childhood and adolescence (see Age-related Changes in EF), cognitive interventions should result in a magnification of age differences and individual differences. In fact, there are a number of earlier studies supporting this account, most of them from the field of memory strategy training, for instance by means of the method of loci (e.g., Björklund and Douglas, 1997; Brehmer et al., 2007; see Verhaeghen et al., 1992 for a meta-analysis).

Second, the compensation account assumes that high-performing individuals will benefit less from cognitive interventions, because they are already functioning at the optimal level and therefore have less room for improvement. Thus, baseline cognitive performance should be negatively correlated with training gains and age differences and individual differences should be reduced after the intervention. Evidence supporting the compensation account comes from numerous studies focusing on EF training, revealing that training-related benefits were larger in children and older adults than in younger adults (e.g., Kramer et al., 1995; Kray and Lindenberger, 2000; Cepeda et al., 2001; Bherer et al., 2008; Kray et al., 2008; Karbach and Kray, 2009; Dorbath et al., 2011). While these studies were based on comparisons at the group level, recent studies also have analyzed correlations between baseline cognitive ability and training-related benefits, indicating that working memory training yielded larger training and transfer effects in children with low cognitive performance at pretest (Jaeggi et al., 2008; Dahlin, 2011; Karbach et al., 2014; but see Loosli et al., 2012; Holmes and Gathercole, 2013).

Moreover, recent work has applied latent variable approaches to analyze individual differences in performance changes as well as correlations between baseline cognitive ability and training-related benefits. One of these studies provided evidence for the magnification account: Loevdén et al. (2012) analyzed data from a study on episodic memory strategy training (based on the method of loci), including children and adolescents (9–12 years) as well as younger adults (20–25 years) and older adults (65–78 years). Even though strategy instructions at the beginning of training reduced individual differences in memory performance, further training ultimately magnified individual differences. In contrast, a study on process-based task-switching training provided evidence for the compensation account (Karbach and Spengler, 2012): children (8–10 years), younger adults (18–26 years), and older adults (62–76 years) consistently showed a reduction of age differences and individual differences not only in terms of performance gains on the training task, but also for transfer to a new, untrained switching. Moreover, cognitive performance at pretest was negatively correlated with training and transfer gains, suggesting that low-performing participants showed larger training-induced gains.

Taken together, research investigating the role of baseline cognitive ability for training-related performance gains indicated that magnification effects were more likely in the domain of strategy training, whereas compensation effects were found more often after process-based training interventions, such as EF training (for further comments on the difference between strategy-based and process-based interventions see Lustig et al., 2009; Noack et al., 2009; Kliegel and Bürki, 2012; Verhaeghen, 2014).

## POTENTIAL FOR THE APPLICATION IN CLINICAL AND EDUCATIONAL SETTINGS

The cognitive and neural plasticity uncovered in the field of cognitive training research certainly has important implications for applied settings, such as clinical or educational programs. At this point, results from well-controlled training studies conducted in these areas are still limited, but the latest basic research findings may be very informative for the design of applied training programs. In order to illustrate the potential of EF training for clinical end educational settings, we briefly review selected research findings on (1) EF training in samples suffering from ADHD and (2) the effects of EF training on academic achievement in childhood and adolescence.

### EFFECTS OF EF TRAINING IN PARTICIPANTS SUFFERING FROM ADHD

Attention-deficit hyperactivity disorder is typically characterized by the three behavioral core symptoms inattention, impulsivity, and hyperactivity (Barkley, 1997). Consistently, children with ADHD usually show cognitive impairments in terms of working memory, inhibitory control, and attention. Many life outcomes are negatively affected by the disorder, such as academic development, vocational success, and social interactions (cf. Shah et al., 2012). It is therefore not surprising that many cognitive training studies have aimed at compensating cognitive and behavioral symptoms and supporting the social and scholastic development of children with ADHD.

One recent study has adopted a task-switching training regime that was effective in healthy individuals. Boys between the ages of 7 and 12 years diagnosed with ADHD and medicated with methylphenidate performed four sessions of intensive task-switching training. Compared to a single-task practice condition, the task-switching training benefited inhibition and working memory, both of which are typically impaired in children with ADHD, but not fluid intelligence (Kray et al., 2012). These findings indicate that even relatively short interventions have the potential to selectively improve cognitive deficits associated with ADHD.

A number of other studies have applied the CogMed training battery that has been designed to improve working memory and executive control^1^ (see also Klingberg et al., 2005). It includes a variety of verbal and visuo-spatial short-term memory and working memory tasks that are usually trained for 25 sessions. Several studies have provided evidence for the effectiveness of CogMed training in children with ADHD (e.g., Klingberg et al., 2002, 2005). After the training, children improved their performances on new untrained working memory tasks, but also on measures of inhibition and fluid intelligence. The authors attributed these findings to increased neural efficiency in overlapping neural circuits that were recruited for performing the training and the transfer tasks (Klingberg et al., 2005). Moreover, these improvements were also observed in terms of parent-rated symptoms of inattention and hyperactivity/impulsivity and many gains were maintained for three months (e.g., Klingberg et al., 2005; see also Beck et al., 2010).

Despite these and other encouraging findings, recent reviews and meta-analyses suggest that the effects of working memory training in children with ADHD may not be that far reaching (Rapport et al., 2013; Chacko et al., 2014). While many interventions resulted in significant improvements on the training task and on structurally similar near transfer tasks, particularly far transfer to untrained cognitive domains, behavioral symptoms, and academic outcomes was not significant. Moreover, a few methodological flaws were criticized, such as the use of non-adaptive or no-contact control groups, the use of individual tasks instead of batteries in order to measure constructs, or the analysis of reports from parents that were not blind to training conditions (Shipstead et al., 2012). In fact, a well-controlled recent study on the effects of CogMed training in 7- to 11-year-old children with ADHD did not show beneficial effects beyond working memory improvements (Chacko et al., 2014; see also Holmes et al., 2009b). Thus, should we consider this type of cognitive intervention ineffective in children with ADHD? As nicely summarized by Gathercole (2014) a recent comment, the answer to this question should definitively be no. Instead, we agree that it will be crucial to overcome methodological issues by designing new approaches yielding functional transfer with relevant cognitive benefits. According to Gathercole (2014), this may for instance be achieved by designing hybrid training protocols including features of different types of training that have been proven beneficial, such as *n*-back training (e.g., Jaeggi et al., 2011) and task-switching training (Karbach and Kray, 2009; Kray et al., 2012). Moreover, she suggested to directly implement adaptive training methods into activities that children with ADHD struggle with in the classroom, such as mental arithmetic, following instructions, and language comprehension.

Another issue that has to be considered is the fact that there usually are large individual differences in the effectiveness of cognitive interventions (see Individual Differences in Training-induced Gains; Titz and Karbach, 2014). This is particularly important in children suffering from ADHD, because there is a large variety of causes for the cognitive and behavioral symptoms, such as genetics, anxiety, life stress, exposure to environmental toxins, etc. (Millichap, 2008; Shah et al., 2012). In addition, children may differ with respect to the treatments they previously received as well as regarding their motivation to comply with the training protocol. These and other factors may very well result in big individual differences in training-induced gains, and these differences may mask large individual gains if data is only analyzed on the group-level (Shah et al., 2012). Unfortunately, many clinical studies have not analyzed individual differences, most likely because the sample sizes were too small. While this certainly is an important issue to deal with in future studies, increasing the sample sizes may also come at a cost: according to Shipstead et al. (2012), many small-scale studies reported effects that were not replicated in larger-scale interventions. However, this may not necessarily indicate that the training is not effective, but may be caused by the difficulty of maintaining the integrity of the exact training protocol in a larger context: “Resource constrains may give researchers a difficult choice: small-scale studies that do not have the sample size to consider the role of individual differences, or larger-scale studies that allow one to assess individual differences but cannot have the same level of experimenter control” (Shah et al., 2012, p. 205).

In sum, more fine-grained analyses of the mechanisms underlying transfer of cognitive training and individual differences therein is clearly needed in order to determine which type of training may be most beneficial for children suffering from ADHD or other neurocognitive or developmental disorders. The present results nonetheless show considerable cognitive and neural plasticity in children suffering from ADHD (Jolles and Crone, 2012; Rapport et al., 2013; Chacko et al., 2014), indicating that the individual benefits of well-tailored cognitive interventions may be considerable.

### EFFECTS OF EF TRAINING ON ACADEMIC ACHIEVEMENT

Research on academic achievement has repeatedly confirmed EF, and particularly working memory, as important prerequisites for the general ability to acquire knowledge and new skills. EF are not only related to higher-level cognitive abilities contributing to academic success, such as problem solving, but also to performance in the classroom (for a review, see Titz and Karbach, 2014). In fact, EF have been shown to explain at least as much variance in academic achievement as intelligence (e.g., Swanson, 2004; Altemeier et al., 2008; Andersson, 2008; Alloway and Alloway, 2010; Lu et al., 2011), which is usually considered the most powerful predictor of academic success (e.g., Gottfredson, 2002; cf. Gustafsson and Undheim, 1996).

Studies investigating the contribution of EF to scholastic achievement have often focused on the domains of language and mathematics and showed that EF are directly associated with math ability as well as with reading, writing, and language comprehension (Titz and Karbach, 2014). The strong association between EF and academic achievement is also supported by findings showing that children suffering from developmental disorders or learning disabilities often display specific EF deficits, suggesting these deficits are risk factors for poor academic performance and development (e.g., Barkley, 1997; Gathercole et al., 2006; Schuchardt et al., 2008; see also Effects of EF Training in Participants Suffering from ADHD). Considering this strong relation between EF and academic abilities, one may assume that even small increases in EF functioning might improve children’s academic performance.

However, despite the growing number of cognitive training studies, only very few of them included transfer tasks from the domain of academic abilities. Most of these studies have applied working memory training regimes to children with cognitive deficits or learning difficulties (for an extensive review, see Titz and Karbach, 2014). This work showed that 25 sessions of CogMed working memory training transferred to new, untrained working memory tasks in children with low working memory ability (8–11 years of age), but not to reading or mathematical reasoning abilities (Holmes et al., 2009a; Dunning et al., 2013). In contrast, a recent field study from the same group showed that teacher administered CogMed working memory training improved performance on standardized tests for English and math in sixth grade (Holmes and Gathercole, 2013), indicating that training-induced memory improvements may transfer to ecologically valid measures of academic achievement in low-achieving students. These findings are supported by results showing that students with special educational needs and attention problems (9–12 years) benefited in terms of reading comprehension and basic number skills (Dahlin, 2011, 2013). In contrast to CogMed training, an interactive working memory training game called Jungle Memory including 32 sessions of verbal and visuo-spatial working memory tasks, yielded no transfer to performance on tasks assessing arithmetic and spelling in children with learning difficulties (mean age = 10.10 years; Alloway et al., 2013).

As for healthy children, two recent studies have applied tasks from the Braintwister working memory training battery (Buschkuehl et al., 2008) that included complex verbal working memory tasks. After 10–14 sessions of training, both studies consistently showed improvements on standardized tests of reading in students between 7 and 11 years of age (Loosli et al., 2012; Karbach et al., 2014). In both studies, the authors attributed the transfer to reading to the strong relation of complex span tasks to reading ability (e.g., Daneman and Merikle, 1996; Engel de Abreu et al., 2011) and memory retrieval (Unsworth and Engle, 2006).

In sum, recent developmental findings indicated that cognitive training might indeed compensate EF deficits in children with ADHD and support school-related abilities and academic performance. However, it is also obvious that these effects are not consistent across studies and it is still unknown to what extent they may be modulated by age-related differences in social and emotional processes or by motivational components. Nevertheless, the existing findings are encouraging because they demonstrate the potential of cognitive training for improving daily life performance outside of the lab, even if much more research is needed to fully uncover the underlying mechanisms and to identify training regimes that reliably and consistently improve specific areas of academic performance and development or specific cognitive deficits in clinical samples.

## CONCLUSION

A large body of research has confirmed the multidimensional structure of EF and the importance of fronto-parietal networks for the integrity and development of executive control. Considering the contribution of EF to various life outcomes, many studies have investigated the effectiveness of cognitive training interventions designed to improve EF. This research showed that cognitive plasticity is considerable across the lifespan, even up to very old age. It has also been suggested that behavioral and neural plasticity are especially high in childhood and the prefrontal lobes are particularly sensitive to environmental influences in that age group. Consistently, research on children and adolescents showed that process-based EF training is an effective means to improve control abilities, particularly working memory and cognitive flexibility. Moreover, many EF trainings benefited performance on tasks that were not trained, such as measures of attention or fluid intelligence, even though other studies suggested that these effects may neither be robust nor consistent. Recent work suggest that they may be (a) increased if the training and the transfer task share overlapping processing components and brain regions and (b) more likely after process-based trainings than after interventions teaching task-specific strategies.

The analysis of individual differences in training-induced gains showed that process-based interventions have yielded compensation effects with larger gains in participants that scored worse at pretest. These findings suggest that process-based trainings may be particularly useful for compensating specific EF deficits associated with neurodevelopmental disorders and learning difficulties. In fact, earlier research on working-memory training and task-switching training resulted in significant training gains and broad transfer effects in children with ADHD, even though recent studies have challenged these optimistic results to a certain degree.

Aside from clinical settings, recent studies have also focused on educational contexts. The few existing studies have provided mixed but encouraging findings, indicating that working-memory training has the potential to improve academic abilities, particularly in the domain of language and reading. These benefits have not only been reported for normally developing children, but also for students with cognitive deficits and learning difficulties. Cleary, further research is needed to improve the understanding of the mechanisms mediating transfer of cognitive training to academic abilities. These studies will be of major importance for tailoring training interventions to the specific needs of certain populations or individuals. Moreover, future studies may want to assess how social and emotional development is related to training-induced improvements and to which degree training-related benefits may be driven by motivational components.

## Conflict of Interest Statement

The authors declare that the research was conducted in the absence of any commercial or financial relationships that could be construed as a potential conflict of interest.

## Footnotes

1 www.cogmed.com
